# Retroelement Insertion in a CRISPR/Cas9 Editing Site in the Early Embryo Intensifies Genetic Mosaicism

**DOI:** 10.3389/fcell.2019.00273

**Published:** 2019-11-08

**Authors:** Jeehyun Jeon, Jung Sun Park, Byungkuk Min, Sun-Ku Chung, Min Kyu Kim, Yong-Kook Kang

**Affiliations:** ^1^Development and Differentiation Research Center, Korea Research Institute of Bioscience and Biotechnology (KRIBB), Daejeon, South Korea; ^2^Department of Animal Science, Chungnam National University, Daejeon, South Korea; ^3^Division of Clinical Medicine, Korea Institute of Oriental Medicine, Daejeon, South Korea; ^4^Department of Functional Genomics, University of Science and Technology (UST), Daejeon, South Korea

**Keywords:** CRISPR, Cas9, embryo, mosaicism, sgRNA, embryonic stem cell, indel depth

## Abstract

Continued CRISPR/Cas9-mediated editing activity that allows differential and asynchronous modification of alleles in successive cell generations expands allelic complexity. To understand the earliest events during CRISPR/Cas9 editing and the allelic selection among the progeny of subsequent cell divisions, we inspected in detail the genotypes of 4- and 8-cell embryos and embryonic stem cells (ESCs) after microinjection of a CRISPR toolkit into the zygotes. We found a higher editing frequency in 8-cell embryos than in 4-cell embryos, indicating that the CRISPR/Cas9 activity persisted through the 8-cell stage. Analysis of a CRISPR/Cas9 transgenic founder mouse revealed that four different alleles were present in its organs in different combinations and that its germline included three different mutant alleles, as shown by the genotypes of the pups. The indel depth, which measured the extent of indels at the sequence level within single embryos, decreased significantly as the embryos advanced to form ESCs, suggesting that exclusion of fatal indels occurred in the subsequent cell generations. Interestingly, we discovered that the CRISPR sites frequently contained introduced retroelement sequences and that this occurred preferentially with certain classes of retroelements. Therefore, in addition to CRISPR/Cas9's innate mechanism of separate, differential enzymatic modifications of alleles, the frequent retroelement insertions that occur in early mouse embryos during CRISPR/Cas9 editing further expand the allelic diversity and mosaicism in the resulting transgenic founders.

## Introduction

The clustered, regularly interspaced short palindromic repeat/CRISPR-associated protein 9 (CRISPR/Cas9) system, once known as the bacterial immune system against invading viruses (Barrangou et al., 2007), has revolutionized genome engineering through its high precision and efficiency (Adli, 2018). The CRISPR/Cas9 editing method has two associating transactors, single-guide RNA (sgRNA) and CAS9 nuclease. A 20-nucleotide sequence within the sgRNA that is complementary to the target DNA sequence confers specificity on CAS9, which as a result creates double-stranded DNA breaks that lead to insertions and deletions (indels) due to imprecise DNA repair through non-homologous end joining (NHEJ). Because of CRISPR/Cas9's extreme flexibility as a genome-editing toolkit, it is possible to target nearly any location in the genome by simply designing a short sgRNA. The ease of use and high efficiency of this method have allowed researchers from diverse fields to employ the CRISPR technology as a method of choice for targeting-based genome modifications (Cong et al., 2013; Jinek et al., 2013; Mali et al., 2013).

However, in the use of CRISPR, several obstacles must be overcome: off-target effects and high mosaicism. The former refers to non-specifically targeted genome modifications that occur due to sequence similarity of the sgRNA to non-specific genomic regions (Fu et al., 2013; Hsu et al., 2013; Pattanayak et al., 2013). This slightly less specific CRISPR genome editing appears to be advantageous to bacteria in that it allows them to cope with viruses that mutate frequently (Adli, 2018). In the study of the genome-wide DNA cleavage specificity of the CRISPR method, ChIP sequencing approaches have been performed, and it has been found that Cas9 off-target binding sites are primarily located at open chromatin loci with mismatches at PAM distal bases (Singh et al., 2015). Reflecting the importance of understanding off-targeting in CRISPR editing, various sequencing technologies that use BLESS (Crosetto et al., 2013), GUIDE-seq (Tsai et al., 2015) and Digenome-seq (Kim et al., 2015) to map the double-stranded breakage sites have been developed. However, mapping of all the CRISPR/Cas9-mediated DNA cleavage and binding sites within the genome is challenging, and these methods may not be completely effective because they depend heavily depend on sgRNA sequences, toolkit delivery methods, and cell type and conditions.

Another obstacle to the use of CRISPR to create gene-edited animals is the high mosaicism that results from the use of this method. In generating transgenic animals, for instance, the CRISPR toolkit is injected into the zygote, and, as it continues to target and cleave the desired gene at different stages during early development, the differential likelihood of editing caused by the uneven distribution of the CRISPR components to the daughter cells gives rise to mosaicism (Aslan et al., 2017; Song et al., 2018; Tadjuidje and Cha, 2018; Mehravar et al., 2019). Therefore, a single session of zygote injection using the CRISPR toolkit results in a variety of mice that carry different alleles with new mutations at the desired locus (Yen et al., 2014). In general, such mosaicism is regarded as an undesirable consequence because the mosaicism, the extent of which is beyond prediction and control, inevitably generates false-positive genotyping results (Oliver et al., 2015) and complicates phenotypic analysis of a transgenic founder (Hashimoto et al., 2016). This makes it necessary to first breed the transgenic founder to obtain F1 progeny and isolate individual mutant strains, a process that takes years in non-rodent species such as non-human primates and livestock animals. Nonetheless, CRISPR-yielded mosaicism has its bright sides, as well. The pool of allelic mutations with different nucleotide sequence changes can easily be expanded to sufficient size to serve as a valuable genetic resource for studying the phenotype, function, dosage effect, and human diseases related to a candidate gene (Yen et al., 2014; Zhong et al., 2015; Markossian and Flamant, 2016; Yasue et al., 2017).

In genome editing in which CAS9 recombinant protein is used to cleave the chromosomal DNA immediately after delivery into the cultured cells, the editing rate reaches a plateau by 24 h post-transfection; this method has much higher efficiency than is obtained by the use of a Cas9 expression plasmid (Kim et al., 2014). The cited study also showed that CAS9 proteins were removed within 24 h after transfection, whereas those expressed from a plasmid persisted for several days. Therefore, recombinant CAS9 protein offers the benefits of lower toxicity and greater effectiveness than Cas9 mRNA from an expression plasmid (Bhattacharya et al., 2015). Assuming that the continuous presence of CAS9 and sgRNA in cells intensifies mosaicism and off-target mutations, in this study we examined the persistency of CRISPR activity (i.e., recombinant Cas9 protein activity) in early mouse embryos by measuring the editing frequency in genome-edited embryos at different developmental stages, including 4-cell and 8-cell embryos and blastocyst outgrowths. We also examined how stably the indels acquired in early cleavage embryos were inherited through cell division by measuring the number and depth of indels in later-stage embryos and ESCs. Finally, we estimated mosaicism in various organs and in the germline of a *Trp53* transgenic founder mouse. Notably, we observed a recurrent insertion of reverse-transcribed endogenous retrovirus (ERV) sequences at the sgRNA target regions by the NHEJ pathway, which imposes an additional layer of allelic complexity and exacerbates the mosaicism in CRISPR embryos. ERVs are a type of transposable element that is abundant in most vertebrates, and they make up ~7~8% of the human genome (Rowe and Trono, 2011; Tokuyama et al., 2018). In most cells, these elements are constantly monitored and tightly regulated to preserve genome integrity (Rowe and Trono, 2011; Gifford et al., 2013). However, they are active for a short period of time during early embryonic development due to global DNA demethylation [14]. Increased amounts of ERV transcripts have been repeatedly reported in early human and mouse embryos and are considered to be necessary for proper embryonic development (Rowe and Trono, 2011; Gifford et al., 2013; Gerdes et al., 2016; Tokuyama et al., 2018; Fu et al., 2019). The mobility of retroelements is a key component of genotypic variation and a source of inter- and intra-individual diversity (Richardson et al., 2014). We believe that our results contribute to an understanding of the earliest events of CRISPR editing that occur in mouse cleavage embryos and the change in the pool of mutant alleles that occurs in subsequent cell divisions; they emphasize the need for a molecular device that deactivates the CRISPR activity at as early a stage as possible to reduce the allelic complexity in the resultant transgenic founder animals.

## Results

**Figure 1:**
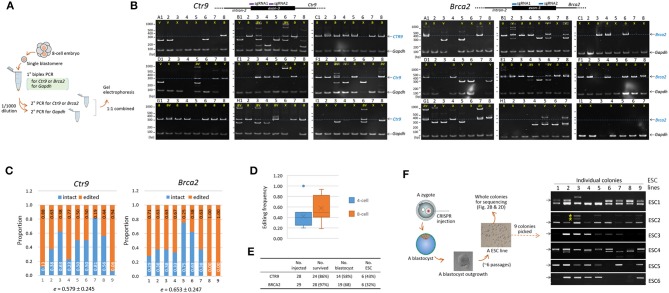
Indel patterns and frequencies in mouse 8-cell embryos and embryonic stem cells derived from CRISP/Cas9-mediated editing of mouse zygotes. **(A)** Strategy for PCR genotyping. Eight-cell embryos derived from the microinjection of sgRNAs and recombinant Cas9 proteins into the pronuclei of mouse zygotes were disassembled to obtain single blastomeres before primary PCR to simultaneously detect the *Gapdh* and either the *Ctr9* or the *Brca2* loci. Nested or hemi-nested PCR (2° PCR) was performed using the primary PCR product, and the resulting product was combined and resolved on a polyacrylamide gel (PAGE). **(B)** Determination of the *Ctr9* (left) and *Brca2* (right) genotypes in individual blastomeres of 8-cell embryos (A to I, *n* = 9) based on the sizes of PCR products. Each PAGE represents a single embryo containing eight blastomeres (numbered 1-8); individual lanes are marked by “v” and “a” based on the types of alleles they contain (indel and normal, respectively). Lanes devoid of *Ctr9* or *Brca2* PCR product are either marked by “x” or unmarked depending on the presence or absence of the *Gapdh* PCR band, respectively. The blastomeres in the *Ctr9* “B” embryo exhibited more PCR bands than would be expected from a diploid cell, hinting at a polyploid (Angell et al., 1987; Munne et al., 1994). The additional PCR bands at higher positions (for instance, those in the B3, B6, D5, F3, F4, H1, H3, and I6 *Ctr9* embryos), especially in the blastomeres carrying heterozygous DNA bands, are heteroduplexes formed between amplicons harboring different indels during PCR (Zhu et al., 2014). The schematic diagram shows the loci at which the two sgRNAs bind. The dotted line on the PAGE image indicates the amplicon position from the wild-type allele. *Gapdh*, internal control. The arrow indicates the position of the PCR band obtained from the wild-type allele. **(C)** Calculation of editing rates (*e*) in the *Ctr9* and *Brca2* CRISPR embryos. The fractions of wild-type alleles (blue) and indel alleles (orange) are calculated based on the number of marks in the lanes **(B)**. **(D)** Box plot for comparison of the *Ctr9* editing rates in 4-cell and 8-cell embryos. Mean values are denoted by X. **(E)** Summary of derivation of embryonic stem cell (ESCs) lines from the *Ctr9* and *Brca2* CRISPR-edited zygotes. **(F)** Genotyping of individual colonies (*n* = 9) in each of the ESC lines (ESC1–6). Some of the ESC5 and ESC6 colonies yielded no amplicons, suggesting that these colonies may originate from the “x”-marked blastomeres that experienced large indels.

To assess the extent of genetic mosaicism in early mouse embryos, we determined the genotypes of individual blastomeres within single mouse embryos. Four- and eight-cell embryos were obtained after microinjection of CAS9 recombinant proteins and a pair of sgRNAs into the zygotes. *Brca2* (breast cancer 2) or *Ctr9* (CTR9 Homolog, PAF1/RNA polymerase II complex component) was chosen as targets because these genes are implicated in the formation of blastocyst outgrowth and embryonic stem cells (ESCs; see below), by which we anticipated to see some change in the mosaicism and the allelic complexity as the cleavage embryos grow to outgrowths and ESCs. For PCR genotyping of single blastomeres, a nested PCR strategy was necessary to secure target amplification (Figure 1A). As an internal control, the *Gapdh* locus was concurrently amplified to ensure the presence of blastomeric genomic DNA.

### CRISPR-Generated Diverse Indels in Early Embryos and Embryonic Stem Cells

Figure 1B shows a variety of indels in the *Ctr9*-edited 8-cell embryos. Indel variants, which are labeled as “v” (as opposed to “a” for the apparently normal allele), were distinguishable even among the blastomeres within single embryos. Some blastomeres displayed the *Gapdh* PCR band but no *Ctr9* bands (these are labeled “x” in embryos A and I, for instance); these embryos were presumed to carry large indels that escaped PCR detection, if the anomaly did not result from experimental errors that frequently occur when a single cell is used as the template in PCR.

We estimated the mutation frequency in single embryos. The editing rate, $e=1-\left(\frac{a}{w}\right),$ where “a” and “w” indicate the counts of normal alleles and whole alleles in single embryos, respectively, varied greatly among the embryos, ranging from 0.188 to 0.938 (0.579 ± 0.245, on average; Figure 1C). Even this estimate seemed to be low considering the possible presence of short indels that were irresolvable on polyacrylamide gels (see below). The editing rate of 4-cell embryos (Supplementary Figure 2) was 0.425 ± 0.234 on average, slightly lower (*p* = 0.180, *t-*test) than that of 8-cell embryos (Figure 1D). The results of genotyping and editing-rate assessment in *Brca2* embryos are also shown in Figures 1B,C, respectively; the editing rate was 0.653 ± 0.247, higher than but not significantly different from that of *Ctr9* embryos (*p* = 0.527, *t*-test). Single-guide RNA-only injection (without Cas9 protein) did not yield any indel at the CRISPR sites, indicating that the *Ctr9* and *Brca2* indels were initiated by Cas9-mediated double strand breaks at the target loci (Supplementary Figure 3). For reference, we additionally provided *Tslp* (thymic stromal lymphopoietin) gene CRISPR result in the Supplementary Figure 2; the mean editing frequency was 0.502 ± 0.232 in 8-cell embryos, indicating that the CRISPR editing rates looked very similar among the three different target loci.

We next examined the indels present in individual colonies of ESCs. Both CTR9 and BRCA2 are implicated in ESC functions; mouse embryos with no BRCA2 function fail to generate ESCs (Ludwig et al., 1997; Suzuki et al., 1997), and CTR9 serves as a component of the PAF1 complex that is involved in the maintenance of ESC pluripotency (Ding et al., 2009) and the lineage specification of cells in blastocysts (Zhang et al., 2013). *Brca2-* and *Ctr9-*edited zygotes were grown to the blastocyst stage and each blastocyst was used to derive a single ESC line through blastocyst outgrowth; in this way, a total of 12 ESC lines (6 from *Ctr9*-edited blastocysts and 6 from *Brca2*-edited blastocysts) were obtained (Figure 1E, see Figure 1F for the whole procedure). Genotyping of nine individual colonies in each *Ctr9* ESC line found various types of indels, as observed in individual blastomeres, but the allelic complexity appeared somewhat reduced (Figure 1F).

### Measurement of Mosaicism and the Depth of Indels in the CRISPR Embryos

**Figure 2:**
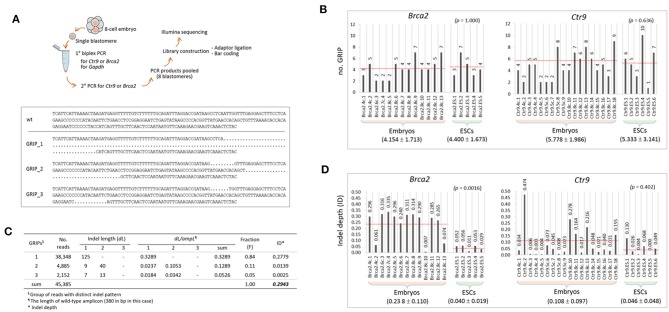
Characterization of the indels in the CRISPR embryos and ESCs at the sequence level. **(A)** Illustration of the procedure for amplicon sequencing using PCR products (top) and a typical result of *Brca2* amplicon sequencing and read alignment for a single sample (bottom). Dots represent deletion of sequence. GRIP, group of reads with distinct indel patterns. Wt, wild-type sequence. **(B)** Counts of groups of reads with distinct indel patterns (GRIPs) in individual samples. The target gene name and the nature of the sample (4-cell, 8-cell, or ESC) are indicated in the sample name. **(C)** An example of calculation of indel depth (*ID*) for the sample in A. Each GRIP has different indel(s), and the indel length (*dL*) of a GRIP is defined as the length of the inserted and deleted sequences relative to the length of the wild-type allele. Fraction (F) of a GRIP denotes the number of reads in the GRIP relative to the number of reads in all the GRIPs of a sample. The *dLs* of the three GRIPs in the sample are 0.3289, 0.1289, and 0.0526; after weighting by the fraction represented by each GRIP, their *ID* values are calculated as 0.2779, 0.0139, and 0.0025, respectively. The sum of these values is defined as the *ID* of the sample and is calculated to be 0.2943. **(D)** Calculation of IDs in individual samples. In **(B,D)**, the mean values of GRIPs and IDs are indicated below the sample groups (±standard deviation; see also the dotted red lines on the graph). Statistics are shown at the upper right in parentheses (2-sample *t*-test).

To inspect the indels at the sequence level, we performed Illumina sequencing of the PCR products used in genotyping, as illustrated in Figure 2A. From sequencing, ~200 million reads (two million reads per sample on average) were obtained, and ~70% of them were aligned to our customized reference (Supplementary File 1). The lower panel of Figure 2A shows an example of the alignment result for a single barcoded sample that was found to possess three groups of reads with distinct indel patterns (GRIPs). The number of GRIPs per sample, which indicates the extent of mosaicism, varied among the samples and ranged from one to 10 (Figure 2B). The mean number of GRIPs in the ESC group (see also Figure 1F) did not differ from that in the embryo group (*p* > 0.636, *t*-test) for either *Brca2* or *Ctr9* CRISPR editing.

We had hoped to determine whether the indel genotypes were stably passed down through cell division, but a longitudinal study using early embryos was not possible. Instead, we measured the extent of an indel (*dL/ampL*) as the length of an indel (*dL*) relative to the length of the wild-type (reference) amplicon (*ampL*), which was weighted by the fraction of the corresponding GRIP in the sample. Given the presence of multiple GRIPs (*n* = *i*, see below) within a single sample and even multiple indels (*n* = *j*) per GRIP, we used the following equation to estimate the indel depth (*ID*) of a sample:

$$\begin{array}{l} ID=\sum F_{i\;}\times {\left(\frac{{\sum dL}_{j}}{ampL}\right)}_{i} \end{array}$$

In this equation, F_*i*_ and ∑*dL*_*j*_ indicate the fraction of reads of the *i*th GRIP and the summed *dL* from the *j* indels in the *i*th GRIP in a sample, respectively. Figure 2C shows the calculation of *ID* (0.2943) of the sample presented in Figure 2A. Calculation of the *ID* values for individual samples showed that they were higher in the embryo group than in the ESC groups for both *Brac2* and *Ctr9* samples (Figure 2D); interestingly, the *ID* difference was significant in the *Brca2* samples (*p* = 0.0016) but not in the *Ctr9* samples (*p* = 0.402). The result suggests that indel inheritance is not secure in cell generations and that there is a process that excludes harmful indels during the progression of embryonic cells to ESCs. The fraction of wild-type reads per embryo sample was lower in the 8-cell embryos than in the 4-cell embryos (43.1 vs. 22.0%; *n* = 9 each). Although the difference was not statistically significant (*p* = 0.240), possibly due to the small number of samples analyzed, the observed difference may indicate that CRISPR editing continues through the 8-cell stage, in agreement with the result shown in Figure 1D.

### Somatic and Germline Mosaicism in a *Trp53* Transgenic Founder Mouse

**Figure 3:**
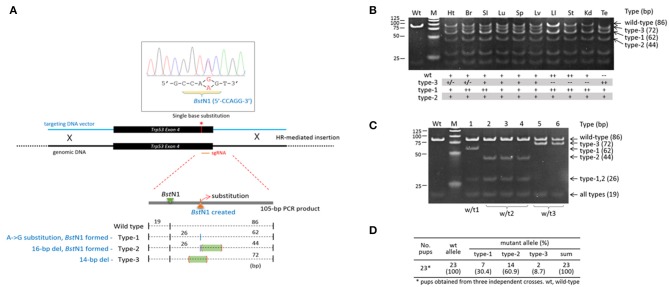
High mosaicism in the organs of a *Trp53*-edited transgenic founder mouse. **(A)** Illustration showing three different types of mutations present at the *Trp53* target site. The 4th exon of the *Trp53* gene was knocked in by homologous recombination using the CRISPR system and a 1.5-kb donor DNA harboring a single-base substitution (red line and asterisk) that creates a new *Bst*N1 recognition site. *Bst*N1 digestion of the 105-bp PCR product flanking the target region discriminates three different types of indel alleles present in a *Tp53* transgenic founder mouse. **(B)** Genotyping of genomic DNAs extracted from transgenic mouse organs by *Bst*N1 digestion of PCR products. Band density is serially graded as “++,” “+,” “+/–,” and “−−.” Note that the wild-type amplicon is absent from the testis. Wt, wild type; Ht, heart; Br, brain; SI, small intestine; Lu; lung; Sp, spleen; Lv, liver; LI, large intestine; St, stomach; Kd, kidney; Te, testis. **(C,D)** Triple mosaicism in the germline of the *Trp53* transgenic founder mouse. Pups were obtained from male *Trp53* transgenic and wild-type female mice and genotyped by *Bst*N1 digestion **(C)**. In total, 23 pups were obtained, and their genotypes were determined **(D)**.

The variability in indels among blastomeres within a single embryo necessarily creates uncertainty regarding the inheritance of complex genotypes by the descendent cells/tissues/animals if the high mosaicism is maintained throughout the development and differentiation of the embryo. If that is the case, those mutant alleles will therefore be ultimately found in the body organs and tissues in various and unpredictable combinations. In order to measure the extent of mosaicism in the organs of a CRISPR mice, we examined a *Trp53* CRISPR knock-in transgenic founder mouse, the tissue genotypes of which could be conveniently determined (also easily readable) by a simple restriction enzyme digestion of PCR products amplified from target locus (Lee et al., 2019). We indeed observed multiple types of mutations in the body of male *Trp53* transgenic mouse: a single-base substitution (type-1), a 16-bp deletion (type-2) and a 14-bp deletion (type-3) at the CRISPR site (Figure 3A). The type-1 and−2 mutations create a new *Bst*N1 site (5′-CCWGG-3′), thus making the two alleles different from the type-3 and the wild-type alleles. Genotyping followed by *Bst*N1 digestion of PCR products revealed that the organs of the transgenic founder contained the mutant alleles in different combinations (Figure 3B): (1) all types of alleles were present in the brain, lung, spleen, and liver; (2) the type-3 allele was rarely present in the heart, large intestine, stomach, or kidney; and finally, (3) the wild-type allele was absent from the testis. We examined the pups born from a cross between the transgenic founder and a wild-type female to observe the germline constitution of the mutant alleles and found all three mutant alleles in the pups (Figure 3C). Each of the mutant alleles appeared at a different frequency among the pups collected from three independent deliveries (Figure 3D). Given that no wild-type pups were born, this demonstrated triple mosaicism and the lack of a wild-type allele in the germline of the *Trp53* founder mouse, in agreement with the testis genotyping result shown in Figure 3B. The result indicates that the organs and tissues of a CRISPR-derived transgenic founder mouse can literally be highly mosaic.

### Frequent Insertions of MT-Int Retroelements at the Ctr9 CRISPR Site

**Figure 4:**
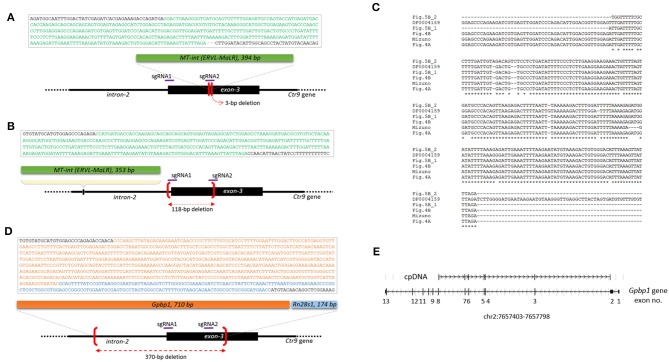
Insertion of reverse-transcribed DNA fragments at the *Ctr9* CRISPR site. **(A,B)** Identification of two independent insertions of MT-int (LTR/ERVL-MaLR) fragments (green box) at the *Ctr9* CRISPR site. The sequences within the box are the MT-int sequences (green) and flanking *Ctr9* sequences (black); deletion regions are indicated using brackets (red). The positions of sgRNAs are indicated by the violet lines. **(C)** Multiple alignment result of the inserted MT-int sequences. The consensus sequence of MT-int (DF0004159; www.dfam.org) is included as a reference. The 394-bp **(A)**, 353-bp **(B)**, 193-bp (Figure 5B_1), 195-bp (Figure 5B_2), and 490-bp (Mizuno et al., 2011) sequences of MT-int inserts are compared. In the Mizuno's paper, 498-bp sequence was reported but the last eight sequence is not aligned with and totally different from the consensus and thus excluded from the sequence alignment analysis (refer to Supplementary Figure 4). **(D)** Identification of complementary sequences of *Gpbp1* (orange, 710 bp) and *Rn28s1* (blue, 174 bp) at the *Ctr9* CRISPR site. The insertion is concurrent with a 370-bp deletion at the *Ctr9* CRISPR locus. **(E)** Mapping of the *Gpbp1* complementary DNA insert (cpDNA) to the genomic *Gpbp1*. The structure of the *Gpbp1* gene and the direction of transcription (arrowheads) are illustrated.

When the PCR amplicons were resolved on a gel, deletion-type mutations were predominant. However, insertion events also occurred at a lower frequency. For instance, the *Ctr9* E6 blastomere shown in Figure 1B yielded an additional PCR band of a larger size. It was identified as a 394-bp MT internal sequence (MT-int) of a non-autonomous class-3 retrotransposon (LTR/ERVL-MaLR; www.dfam.org) (Figure 4A). Another case of insertion was found in the *Ctr9* ESC2 sample, in which two additional large PCR bands (842 and 1,123 bp in size) were present (Figure 1F). Sequencing of the 842-bp fragment identified a 353-bp MT-int sequence plus the *Ctr9* target region with a 118-bp deletion (Figure 4B). The two MT-int inserts found in different embryos were similarly aligned to the 3′ end of the 1,098-bp MT-int consensus (DF0004159; Figure 4C). Mizuno et al. (2011) reported of a spontaneous mutation in *Fgf5* gene due to a transposable element insertion and, interestingly, the end of this inserted sequence, which was later identified to be an MT-int fragment (Gagnier et al., 2019), was exactly the same to our cases (Figure 4C and Supplementary Figure 4 for full alignment result), suggesting the operation of a similar MT-int insertion mechanism using the same reverse-transcribed DNA end. The other 1,123-bp PCR product was found to contain two unrelated DNA fragments: *Gpbp1* (GC-rich promoter binding protein-1; 710 bp) and *Rn28s1* (28S ribosomal RNA; 174 bp) fragments plus the *Ctr9* target locus with a large deletion (Figure 4D). The *Gpbp1* sequence represented the part of the complementary DNA of *Gpbp1* mRNA that covers exons 8 to 2 of a total of 13 exons and lacked intronic sequences (Figure 4E). Given that no *Gpbp1*-related pseudogene was found in the mouse genome (BLAT search), we assumed that the complementary *Gpbp1* fragment was derived from reverse transcription in the early embryo. The *Rn28s1* fragment was aligned to the 3′-end of the gene and multiply mapped to four chromosomal loci as well as to the *rDNA* cluster. Notably, the *Rn28s1* copy on chromosome 14 was flanked by MERVL retroelements (Supplementary Figure 5); this may provide a possible mechanism for *Rn28s1* expression that can be synchronized with MERV transcription in the early embryo.

**Figure 5:**
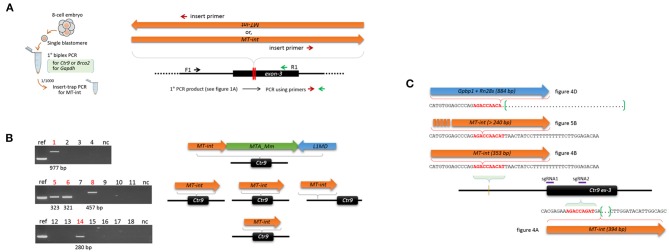
Insert-trap PCR detects MT-int insertion events at the *Ctr9* CRISPR site. **(A)** Schematic showing the insert-trap PCR strategy for finding the 3′-end of an MT-int insert expected at the *Ctr9* CRISPR locus. There are two possible orientations of MT-int DNA insertion, forward and reverse; the use of the red (insert primer) and green (R1) primer set detects the forward MT-int insert only. **(B)** High frequency of MT-int DNA insertion at the *Ctr9* CRISPR site. PCR was performed using as templates the primary PCR products (see Figure 1A) that failed to produce relevant amplicons in the subsequent nested PCR. PCR products of different sizes generated by insert-trap PCR are shown along with the reference amplicon of the sample in Figure 4A (left). The identities and structures of the inserts are schematically represented after sequencing (right). **(C)** Similarity in DNA sequence at the insertion sites. The sequence 5′-AGACCA-3′ appears frequently at the insertion sites. The green brackets and dots indicate deletion regions.

As shown in Figure 1B, some of the *Ctr9* blastomeres failed to show PCR products, although they had the *Gapdh* band. On the assumption that some of these blastomeres captured MT-int sequences at the CRISPR site but PCR failed to detect these inserts because of their large sizes, we used the insert-trap PCR strategy (Kang et al., 1999), which finds the 3′ ends of certain inserts at the target locus (Figure 5A). Insert-trap PCR using the primary PCR products from individual blastomeres (see Figure 1A) as template indeed detected amplicons of various sizes in a portion (27.8%, 5/18) of the blastomeres that were otherwise hidden in the ordinary PCR (Figure 5B). Sequencing identified these as the MT-int sequences, as expected. As illustrated in Figure 5B, all the events except one were simple insertions of an MT-int fragment; in one case, the insert was composed of MT-int, MTA-Mm, and LINE1 sequences. We found that this composite sequence actually exists in the mouse genome at chr17:52426124 (mm10 reference), suggesting that the insert was derived from a read-through transcript of the chimeric sequence. Meanwhile, the Insert-trap PCR detected no band in the blastomeres from wild-type embryos or sgRNA only injected embryos (data not shown), indicating that the appearance of retroelement fragments at the target site depends on the CRISPR editing.

In addition, we found a site, 5′-AGACCAACAT-3′, at which MT-int insertion was frequently observed. This site is located ~250 bp upstream (intron-2 of the *Ctr9* gene) of the sgRNA1 site, and the three different insertions were associated with this sequence (Figure 5C). Notably, the insertion of 394-bp MT-int (see Figure 4A) occurred in a similar sequence context, 5′-AGACCAGAT-3′, which sits on the boundary of the sgRNA2 site in exon-3. This observation hints that the MT-int sequences may exhibit some sequence preference in inserting themselves into the genome. Together, these results indicate that the frequency of large DNA insertion during genome editing is not low and that those inserts are mostly of retroelement origin.

### Preferential Insertion of MERVL**_**2A Sequences at the *Trp53* CRISPR Site

**Figure 6:**
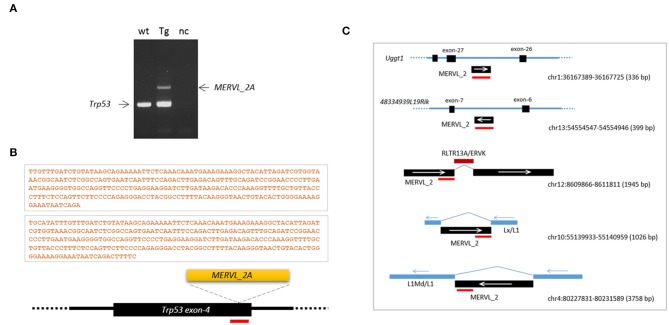
Frequent insertions of MERVL_2A fragments at the *Trp53* CRISPR site. **(A)** Insertion of a MERVL_2A fragment at the CRISPR site in the *Trp53* transgenic founder mouse (see Figure 3). PCR genotyping of tail genomic DNA yielded an additional band that was identified by sequencing as the MERVL_2A sequence. wt, wild-type genomic DNA; Tg, transgenic; nc, negative control. **(B)** Schematic of MERVL_2A insertions and their 280-and 293-bp sequences identified from *Trp53* CRISPR embryos. The red bar indicates the CRISPR site. **(C)** Genomic MERVL_2A copies exist as truncated forms with the potential to produce reverse-transcribed DNAs equivalent in size and sequence to the observed inserts. The red bars indicate the positions of the MERVL_2A insert sequence in individual genomic copies. The arrows denote the direction of transcription of the indicated sequences.

Retroelement insertion was not limited to the *Ctr9* CRISPR site. From PCR genotyping of the tail DNA of the *Trp53* transgenic founder, we unexpectedly observed an additional DNA fragment, which was identified as a MERVL-int (MERVL_2A sequence; DF0001919; Supplementary Figure 6), another LTR/ERVL subfamily present in the mouse genome (Figure 6A; see also Figure 3A). To verify that the entrapment of a retroelement copy at the *Trp53* CRISPR site was not a fortuitous event, we produced *Trp53* CRISPR embryos at the 4- or 8-cell stage using the same sgRNA but no knock-in vector (see Figure 3A) and explored retroelement insertion in those embryos. PCR genotyping detected two embryos (2/22) that yielded larger PCR bands that were identified by sequencing to be, interestingly, the same MERVL_2A sequences (Figure 6B). These MERVL_2A inserts were found to be similar in length (280 bp and 293 bp). Inspection of the mouse genome for the MERVL-int sequences found that some copies indeed exist as truncated or interrupted fragments that can yield reverse-transcribed products equivalent in size and nucleotide sequence to the observed inserts (Figure 6C). We guess that the reverse-transcribed product derived from some full-length MERVL_2A copies may be somehow preferentially cleaved at certain positions and these cleaved products may be occasionally inserted into the CRISPR site. If the predominance of the MT-int sequences at the *Ctr9* CRISPR site is taken into consideration, the MERVL_2A sequences can be regarded as the sequences favored by the *Trp53* CRISPR site. This hints at a preference of certain CRISPR sites for specific retroelements. In summary, our findings indicate that retroelement insertion occurs frequently during CRISPR editing in early mouse embryos. This causes allelic diversity and mosaicism to expand even further in CRISPR embryos.

## Discussion

Zygote injection for CRISPR/Cas9-mediated editing generates multiple mutations in single embryos and increases the allelic complexity at the desired locus. The higher editing rate (Figure 1D) and the lower fraction of the wild-type allele (43.1 vs. 22.0%) in 8-cell embryos compared with 4-cell embryos (Figure 1D) indicate the ongoing action of the CRISPR machinery at the 8-cell stage. The persistence of CRISPR activity to this late cleavage stage hints that the genome editing that occurs during CRISPR editing may not be very processive because a site can no longer be a target once it has been edited at an earlier stage. This low processivity of the CRISPR machinery and its prolonged duration of action increase the pool of allelic mutations in a single individual and inevitably result in higher mosaicism. This mosaicism may increase even further if the mutations are tolerated by the embryo or fetus, as shown by the presence of many different coat-color phenotypes among the *Tyr* CRISPR transgenic founder mice (Yen et al., 2014). When the currently available CRISPR method is used, subsequent studies such as phenotypic analysis of transgenic founder mice can be complicated. To avoid such complexity, certain molecular devices that can improve the processivity of Cas9 and/or limit Cas9 protein activity to a narrow developmental window and then deactivate its function in a timely manner are necessary; such devices might include, for instance, the use of anti-CRISPR proteins that inhibit Cas9-mediated gene editing (Pawluk et al., 2016; Dong et al., 2017; Harrington et al., 2017; Rauch et al., 2017).

The frequency of indels in CRISPR editing may rely on the innate editing power (or the affinity for the target locus) of the sgRNA sequence and the structural openness of chromatin at the target locus. Therefore, the sgRNA sequence itself and the accessibility of its target chromatin can determine the indel rate and the pool size of indels. The editing rate shown in Figure 1C considered the frequency of indels only; it was obtained simply by estimating the proportion of mutant alleles in single embryos, regardless of the length or the severity of the indels themselves. However, the *ID* calculation (Figure 2D) was designed to characterize the indels at the sequence level. It estimates the sizes of individual indels and their proportional representation in single embryos using amplicon reads from deep sequencing. The ID equation has the drawback that it only considers the physical aspects of indels. The genetic nature of a mutation, such as whether it is in or out of frame or is a missense or a non-sense mutation, is not considered by the equation, although it is unambiguously useful to include such genetic aspects of an indel in the calculation to permit assessment of the severity of the mutation in relation to molecular and biochemical function. However, PCR and sequencing errors are not rare, and the error rate may increase greatly when samples containing limited amounts of genomic material, such as early embryos, are used (Min et al., 2017, 2018a). We indeed observed single base-pair insertions, deletions, and substitutions that randomly appeared among the amplicon reads, and we excluded these uncertain single-nucleotide variations (SNVs) from the ID calculation. Therefore, for a new calculation considering the genetic and biochemical aspects of indels to be useful, we should wait for technological improvements in PCR and sequencing that permit only a negligible amount of errors or use a bioinformatics platform to distinguish actual indels from PCR/sequencing errors.

In the CRISPR editing of *Brca2*, the ID value of the ESC samples was significantly lower (~6-fold) than that of the embryo samples (Figure 2D). This indicates that the mean ID value decreases as early embryos proceed to blastocyst outgrowths and ESCs, suggesting that embryonic cells that harbor detrimental indels within the *Brca2* genes, including out-frame and non-sense mutations, fail to survive during this time. The result is consistent with the observation that BRCA2 plays an essential role in the establishment of an ESC line from a single blastocyst (Ludwig et al., 1997). At the molecular level, BRCA2 functions in DNA repair (Roy et al., 2011), and the removal of BRCA2-depleted cells on the path to ESC establishment may increase the sensitivity of ESCs to genome instability and cell cycle arrest (Rocha et al., 2013). Hence, our result is consistent with an indispensable role of BRCA2 in ESCs and suggests that BRCA2 is not essential during the cleavage stage in mice when cell division occurs through an autonomous mechanism. The comparison of ID values, therefore, can help predict how susceptible certain cell types are to CRISPR/Cas9-mediated editing of a target gene that works in a spatiotemporal fashion during development.

We observed the insertion of LTR sequences and, less frequently, other reverse-transcribed gene transcripts at the *Ctr9* and *Trp53* genome editing sites. The actual frequency of DNA insertion is expected to be higher than the observed frequency because there are limitations on the detection of a large insert by PCR genotyping of a single blastomere. Additional MT-int insertions that were not revealed by conventional PCR were unearthed by the use of insert-trap PCR (Figure 5F), which mimics the previously used Hord-PCR (homologous recombination-detection PCR), to identify the flanking sequences around the inserted repeat elements (Kang et al., 1999). Since the method was designed to detect only inserts in the forward direction, if we consider the insertions of MT-int in both directions and the sequences of retroelements of other classes, the actual insertion frequency at the CRISPR site may exceed the observed frequency. Notably, Ono et al., inspected 57 CRISPR transgenic mice and unexpectedly found de novo insertions in 20 of them (Ono et al., 2015); 30 insertions were characterized and 16 insertions were derived from LTR sequences such as MaLR and MERVL while the remained 14 from exonic sequences. Since that study analyzed tissue DNA (tail-tip) of transgenic offspring, instead of early embryos, the DNA insertion frequency at the CRISPR site might be underestimated due to somatic mosaicism for the insertions among the tissues. Our result obtained from whole embryos could be, therefore, considered to be more relevant to estimate the actual insertion frequency occurring at the CRISPR site and, in relation to the Ono's study, proved that CRISPR-mediated DNA insertions found in the transgenic mice are mostly established in early development. Additional line of evidence supporting our result is that from the zygote to the 4-cell stage, MT-int transcripts are the transcripts that are most abundantly represented among the retroelement families (Ge, 2017). It should be also noted that most of the LINE1 copies in the genome are truncated and are thus devoid of a 5′-UTR core regulatory sequence, whereas most ERV LTRs possess their natural transcriptional and regulatory signatures (Rebollo et al., 2012). Therefore, ERVs are certainly the greater source of reverse transcriptions of ERV-derived sequences (Gerdes et al., 2016), particularly during early embryogenesis when genome-wide demethylation and derepression of various retroelements occur (Reik et al., 2001; Surani, 2001). Interestingly, the promoters of ERVs, particularly those of the ERVL family, are known to be involved in the regulation of a group of genes that acts specifically at the 2-cell stage of embryonic development in mice (Macfarlan et al., 2012). Therefore, if a certain CRISPR site acts as a local sink that attracts floating nucleic acids in the nucleus, the ERVL sequences, due to their abundance, could be the sequences most likely to be drawn toward the sink. In agreement with this, both the MT-int and the MERVL_2A sequences that were inserted at the *Ctr9* and *Trp53* CRISPR sites, respectively, belong to the ERVL family. This class-III LTR family is transcribed at extremely high levels, accounting for ~3% of total transcripts at the 2-cell stage in mice (Kigami et al., 2003). In reality, it was shown that, differing from early embryos, NIH3T3 culture cells captured, instead of MERVs, LINE1 sequences most frequently (26% frequency among the captured sequences) at the CRISPR target site (Ono et al., 2015). In addition, we analyzed a public CRISPR dataset [GSE57283; (Frock et al., 2015)] and found that the insertion frequency was quite different between the cell lines; 293T cells were much higher than A549 cells (0.326 vs. 0.009% of total ~1.2 × 10^6^ reads; *p* = 0.018, *t* test), and that LINE1s were found as the primary inserts at the target locus (Supplementary Figure 7). LINE1 sequence was not detected as insert in early embryos in our study (Figures 4, 6) as well as Ono's (2015). We assume that the differences in the frequency of insertion and species of incorporated retroelements might mirror their cell type-specific expression levels.

We assume that retroelement insertion occurs during NHEJ following a double-strand DNA break at the CRISPR site. Although retroelement insertion likely involves a series of concurrent processes of DNA breakage/deletion followed by insertion at the same spot, it may not always occur in this manner. As shown in Figures 4B,D, 5B, there were distinctive insertions that appeared to be unrelated to the CRISPR event because the insertion locus was distant (~250 bp upstream of) from the sgRNA site. This insertion locus is 5′-AGACCAACAT-3′ in the DNA sequence, and, given the repeated insertions at this distant site, they cannot be accidental. The sequence 5′-AGACCAACAT-3′ might be innately associated with the MT-int sequence because a very similar sequence (5′-AGACCAGAT-3′) was found at the insertion site of the 394-bp MT-int insert near the sgRNA2 site within exon-3 (Figure 5C). The same locus in the *Ctr9* alleles of wild-type blastomeres did not show evidence of MT-int insertion (*n* = 46; data not shown), implying that the locus is not a natural site of insertion of MT-int sequences and that the insertion is associated with the CRISPR event. The result suggests the possibility that the surroundings of the CRISPR locus, which may occupy several hundred bases, can be a bed for the integration of retroelements. The idea that MT-int insertion is associated with the DNA sequence around the CRISPR site and that this *Ctr9* CRISPR site favors MT-int sequences is supported by the preferential detection of MERVL_2A sequences at the *Trp53* CRISPR locus (Figure 6), an observation that hints at a preference of specific retroelements for certain CRISPR sites. However, CRISPR sites do not always capture retroelements, and the retroelement insertions and their frequencies may therefore be very dependent on the target genomic loci. We were unable to find any insertion events at the *Brca2* or the *Tslp* CRISPR sites. Our results indicate that the retroelement frequently inserts into a target locus during genome editing in early mouse embryos and that certain retroelement sequences are favored at certain genome editing sites. The insertion of retroelements at the genome editing site exacerbates mosaicism by adding another layer of allelic complexity to the resulting CRISPR embryos.

## Materials and Methods

### Collection of Fertilized Eggs

Mouse zygotes were collected from super-ovulated BCF1 (C57BL/6 × CBA/CA) females as described previously (Hogan, 1994). Briefly, female BCF1 mice at 5 weeks of age were injected with 5 IU of pregnant mare serum gonadotrophin (PMSF), followed by 5 IU of human chorionic gonadotropins (hCG) 48 h apart, and mated with male mice. Successful mating was determined the following morning by detection of a vaginal plug. Mouse zygotes were transferred to M2 medium (Sigma) containing 0.1% (w/v) hyaluronidase to remove cumulus cells and cultured in M16 medium (Sigma) at 37°C, 5% CO_2_ in air (Min et al., 2018a).

### Microinjection of CRISPR Toolkit and Production of *Trp53* Transgenic Knock-In Mouse

Single-guide RNAs (sgRNAs) and recombinant Cas9 protein were purchased from ToolGen. Following is the sequence information of target sites: 5′-GCTTGTAGTATTCCAGCTTTAGG-3′ and 5′-ATGTATCCAAGCAAGTCATCTGG-3′ for *Ctr9* sgRNA-1 and−2, respectively; 5′-TAGGACCGATAAGCCTCAATTGG-3′ and 5′-AGTTGAAGCAAACTGATGGTAGG-3′ for *Brca2* sgRNA-1 and−2, respectively; 5′-AGTTGAAGCAAACTGATGGTAGG-3′ and 5′-TGCAAGTACTAGTACGGATGGGG-3′ for *Tslp* sgRNA-1 and−2, respectively; 5′-CACCGTGCACATAACAGACTTGG-3′ for *Trp53* sgRNA. Both sgRNAs and Cas9 protein were mixed using microinjection buffer (0.1 mM EDTA/10 mM Tris-Cl, pH 7.4) to the final concentration of 40 ng/μl each and incubated for 15 min at 37 °C immediately before microinjection. Microinjection was performed 24 h after human chorionic gonadotropin injection (Kang et al., 1999). To visualize the pronuclei, cumulus-free zygotes were centrifuged at 13,000 rpm for 5 min. Embryos with clearly visible pronuclei were selected, and the mixture of sgRNA and Cas9 were injected into the male pronucleus. Microinjection was performed under an inverted microscope equipped with micromanipulator and a microinjector (Leica). The injected zygotes were cultured in M16 media for 48 h and the resulting 4- and 8-cell stage embryos were collected for genotyping analysis. For production of *Trp53* CRISPR knock-in transgenic founder mouse, the manipulated zygotes were transferred into the oviduct of a pseudopregnant foster mother (C57BL/6) immediately after injection of CRISPR toolkit plus a knock-in vector. To prepare the knock-in targeting vector, 1.4-kb *Trp53* genomic region spanning from the intron-1 to intron-4 and carrying *Trp53* c.350A>G mutation was synthesized and then cloned into pUCIDT plasmid using *Dra*I restriction enzyme site (Cosmogenetech). This donor template DNA was eluted after *Dra*I digestion of the pUCIDT plasmid and dialyzed for purification before microinjection (4 ng//μl) along with the CRISPR toolkit. The tail genomic DNAs were extracted from resultant pups, genotyped by PCR (for primers, see Supplementary Figure 1) and *Bst*N1 enzyme (NEB) digestion, and run on polyacrylamide gel.

### Derivation of Embryonic Stem Cells

The zygotes microinjected with the cocktail of sgRNAs and Cas9 protein were cultured in a CO_2_ incubator to the blastocyst stage. Blastocysts were co-cultured with mouse embryonic fibroblasts (MEF) plated in advance on a gelatin-coated culture dish for 10-14 days in embryonic stem cell (ESC) medium (knock-out DMEM supplemented with 10% knock-out serum replacer, 5% FBS, 0.1 mM NEAA, 2 mM Glutamax, and 0.055 mM β-mercaptoethanol). The derivation of MEFs was described in detail elsewhere (Kwon et al., 2015). The outgrown colonies were picked and treated with 0.05% Trypsin-EDTA for 5 min to dissociate into single cells and were allowed to separately grow to ESC colonies in a dish preplated with MEFs (Dodge et al., 2004). Nine colonies in each ESC line were picked for PCR genotyping at ~6 passages, or the whole colonies were pooled for Illumina sequencing.

### Embryo Biopsy and Genotyping Analysis

For assessment of mosaicism in each embryo, the 4- and 8-cell embryos were individually pretreated with 7.5 μg/ml Cytochalasin B in M2 media for 10 min and carefully physically separated the blastomeres using the injection pipette. After washing with PBS containing 0.1% polyvynylalcohol (PVA), each of the separated blastomeres was transferred into a PCR tube and stored at −20°C. For genotyping, each of the blastomeres was lysed in embryo lysis buffer (ELB: 20 mM Tris-Cl (pH 8.0), 10% Tween 20, 10% Nonidet P-40 and 10 mg/ml proteinase K) at 50°C for 30 min and boiled at 95°C for 10 min before PCR (Kang et al., 2001). PCR was performed using AccuPower PCR PreMix (Bioneer) in the following conditions: the denaturation step of 95°C/3 min, 25 cycles of 95°C/30 s, 55°C/30 s, 72°C/1 min, and the final extension step of 72°C/5 min for the primary PCR, and the same conditions using one-tenth volume of the primary PCR product as template for the hemi-nested PCR. The primer information is in the Supplementary Figure 1. The internal primer used to detect MT-int inserts in the insert-trap PCR was 5'-TGGAGCCCACAGTTAAGAGA-3'. PCR products were resolved on a 5 or 8% polyacrylamide gel.

### Genotyping by High-Throughput Sequencing and Data Analysis

Using amplicons produced for genotyping for Sanger sequencing, Illumina high-throughput sequencing libraries were generated as previously described (Min et al., 2018b, Park et al., 2017). One hundred nanogram of each amplicon library was incubated with T4 polynucleotide kinase (PNK, NEB) at 37°C for 30 min and then ligated with Illumina sequencing adapters. The ligates were amplified using P5 and barcoded P7 primers using following PCR condition: 98°C for 15 min, 20 cycles of 98°C for 20 s, 68°C for 30 s, 72°C for 1 min, and final extension at 72°C for 5 min. Paired-end (250 bp) sequencing was performed using the purified NGS libraries in the MiSeq system (Illumina).

Raw sequencing reads were groomed to remove the adapter and low quality bases using “Trim_galore” (https://www.bioinformatics.babraham.ac.uk/projects/trim_galore/). Since each read was too short to cover the whole amplicon, overwrapping sequences of each read pairs waere merged to generate full-length amplicon sequences using “PANDAseq” [(Masella et al., 2012); https://github.com/neufeld/pandaseq]. The merged reads were aligned on wild type amplicon sequences by “ncbi-blastn” (https://blast.ncbi.nlm.nih.gov/Blast.cgi?PAGE_TYPE=BlastDocs&DOC_TYPE=Download). For INDEL assessments, frequency of reads with unique INDEL patterns were quantified by compiling hits and gaps information in “blastn” results using a home-made bash script.

To explore retroelement insertions in a public dataset, six of the raw FASTQ files (3 × 293T, 3 × A549; SRR1569825-SRR1569830 of GSE57283 dataset) that contain the CRISPR/Cas9 edited RAG1 (RAG1B) sequences (Frock et al., 2015) were downloaded. The reads were groomed to remove the Illumina adapter sequences and low quality bases using “Trim_galore.” Overlapping sequences of each pair of the Miseq 250 bp mates were merged by “PANDAseq” (Masella et al., 2012) with default parameters to generate a longer DNA fragment sequence. First, to verify the genomic origins of the merged DNA fragment sequences, each fragment was aligned by “BLAT” (Kent, 2002) with default parameters to 50 bp downstream genomic region from the primer site the authors used, and only the reads with <3 mismatches were extracted. Next, the fragments were aligned onto the full length transposable elements (TEs) using “BLAT” with default parameters. Finally, the reads were grouped by types of TE family and the number of reads mapped on each TE family was counted.

## Author Summary

The CRISPR/Cas9-mediated editing technology has revolutionized genome engineering. It creates double-stranded DNA breaks that lead to insertion and/or deletion mutations due to imprecise DNA repair through non-homologous end joining (NHEJ). Because of CRISPR's extreme flexibility as a genome-editing toolkit, it is possible to target nearly any location in the genome by simply designing a short sgRNA, and its ease of use and high efficiency have allowed researchers from diverse fields to employ the technology as a method of choice for targeting-based genome modifications. An obstacle to the use of CRISPR to create gene-edited animals is the high somatic and germline mosaicism, which is unpredictable and uncontrollable and complicates phenotypic analysis of a transgenic founder. In this study, we report that certain CRISPR-edited sites frequently contained introduced retroelement sequences and that this occurred preferentially with certain classes of retroelements. We, therefore, believe that in addition to CRISPR's innate mechanism of separate, differential enzymatic modifications of alleles, the frequent retroelement insertions in early mouse embryos during CRISPR/Cas9 editing further expand the allelic diversity and mosaicism in the resulting transgenic founders.

## Data Availability Statement

The data generated in this study is publicly accessible in the Gene Expression Omnibus (accession GSE136393).

## Ethics Statement

This study was carried out in strict accordance with the recommendations in the Guide for the Care and Use of Laboratory Animals of the National Livestock Research Institute of Korea. The protocol was approved by the Committee on the Ethics of Animal Experiments of the Korea Research Institute of Bioscience and Biotechnology. All surgery was performed under sodium pentobarbital anesthesia, and all efforts were made to minimize suffering.

## Author Contributions

JJ: collection and/or assembly of data, sample preparation, PCR genotyping, murine experiments, and data interpretation. JP: zygote preparation/microinjection, embryo culture, blastomere isolation, transgenic production, and other murine experiments. BM: amplicon library construction, sequencing read mapping, and indel classification. S-KC: *Trp53* knock-in vector construction and mutation identification. MK: conception/design and data interpretation. Y-KK: conception, experiment design, data interpretation, statistical analysis, sequencing data analysis, and manuscript writing.

### Conflict of Interest

The authors declare that the research was conducted in the absence of any commercial or financial relationships that could be construed as a potential conflict of interest.
