# Supplementary material for: Retroelement Insertion in a CRISPR/Cas9 Editing Site in the Early Embryo Intensifies Genetic Mosaicism
Source: Front Cell Dev Biol. 2019 Nov 8;7:273. doi: 10.3389/fcell.2019.00273 (PMC6857330; doi:10.3389/fcell.2019.00273)
Supplement: Supplementary file 1 [file Presentation_1.pptx]

## Slide 1
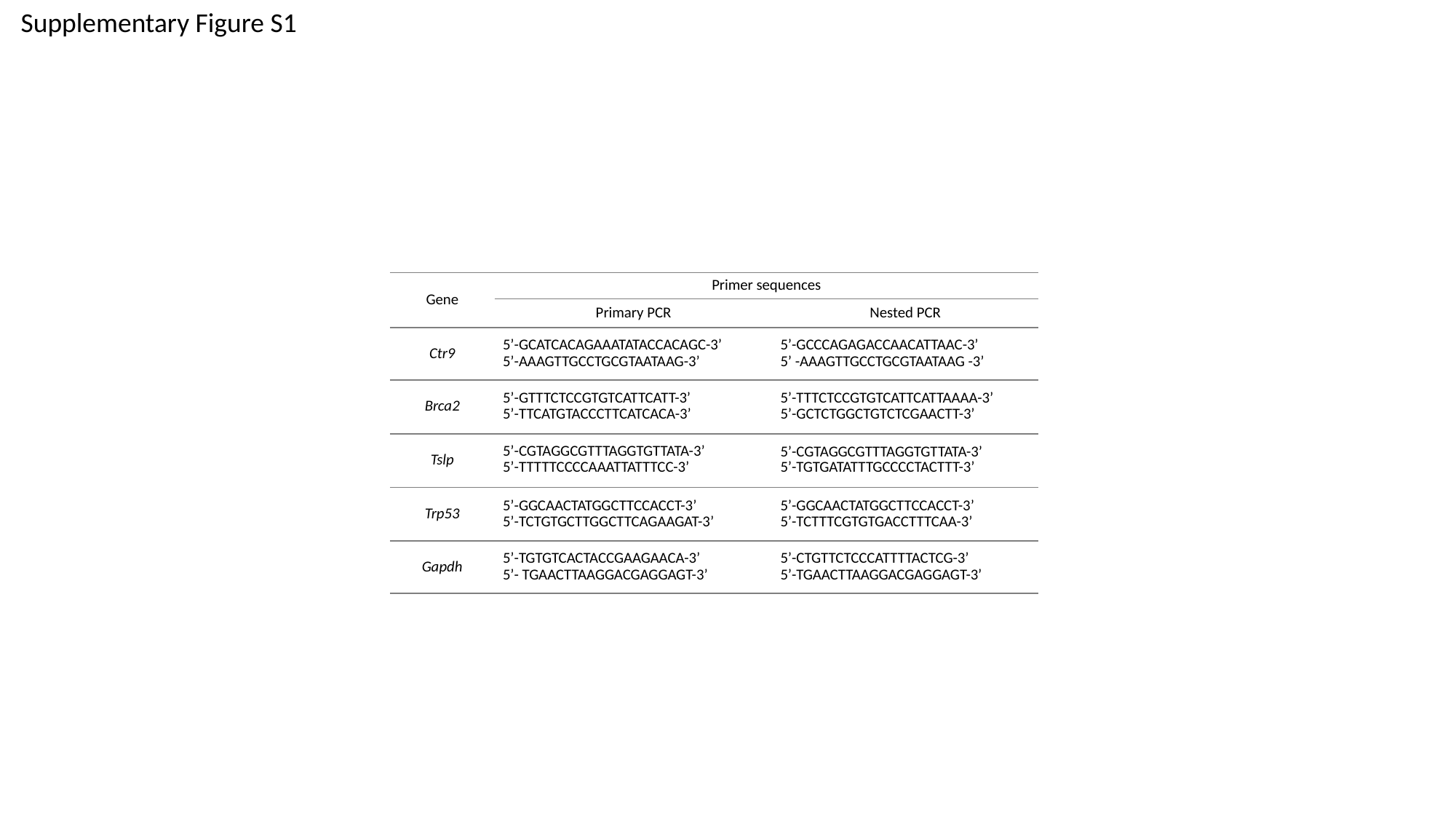

Supplementary Figure S1
| Gene | Primer sequences | |
| --- | --- | --- |
| | Primary PCR | Nested PCR |
| Ctr9 | 5’-GCATCACAGAAATATACCACAGC-3’ 5’-AAAGTTGCCTGCGTAATAAG-3’ | 5’-GCCCAGAGACCAACATTAAC-3’ 5’ -AAAGTTGCCTGCGTAATAAG -3’ |
| Brca2 | 5’-GTTTCTCCGTGTCATTCATT-3’ 5’-TTCATGTACCCTTCATCACA-3’ | 5’-TTTCTCCGTGTCATTCATTAAAA-3’ 5’-GCTCTGGCTGTCTCGAACTT-3’ |
| Tslp | 5’-CGTAGGCGTTTAGGTGTTATA-3’ 5’-TTTTTCCCCAAATTATTTCC-3’ | 5’-CGTAGGCGTTTAGGTGTTATA-3’ 5’-TGTGATATTTGCCCCTACTTT-3’ |
| Trp53 | 5’-GGCAACTATGGCTTCCACCT-3’ 5’-TCTGTGCTTGGCTTCAGAAGAT-3’ | 5’-GGCAACTATGGCTTCCACCT-3’ 5’-TCTTTCGTGTGACCTTTCAA-3’ |
| Gapdh | 5’-TGTGTCACTACCGAAGAACA-3’ 5’- TGAACTTAAGGACGAGGAGT-3’ | 5’-CTGTTCTCCCATTTTACTCG-3’ 5’-TGAACTTAAGGACGAGGAGT-3’ |

## Slide 2
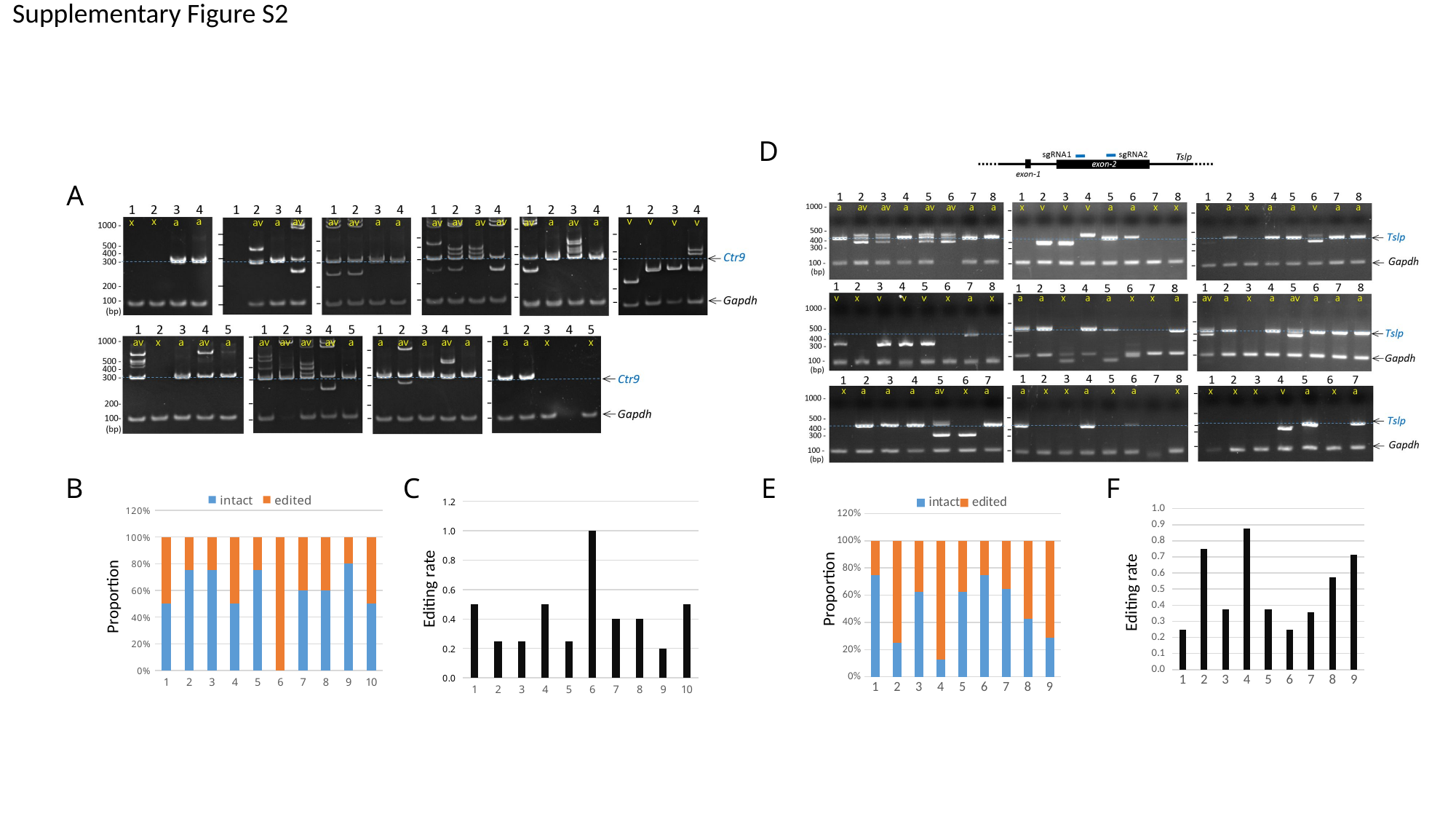

Supplementary Figure S2
D
A
### Chart
| Category | intact | edited |
|---|---|---|B
C
E
F
### Chart
| Category | |
|---|---|
### Chart
| Category | intact | edited |
|---|---|---|Proportion
### Chart
| Category | |
|---|---|Editing rate
Editing rate
Proportion

## Slide 3
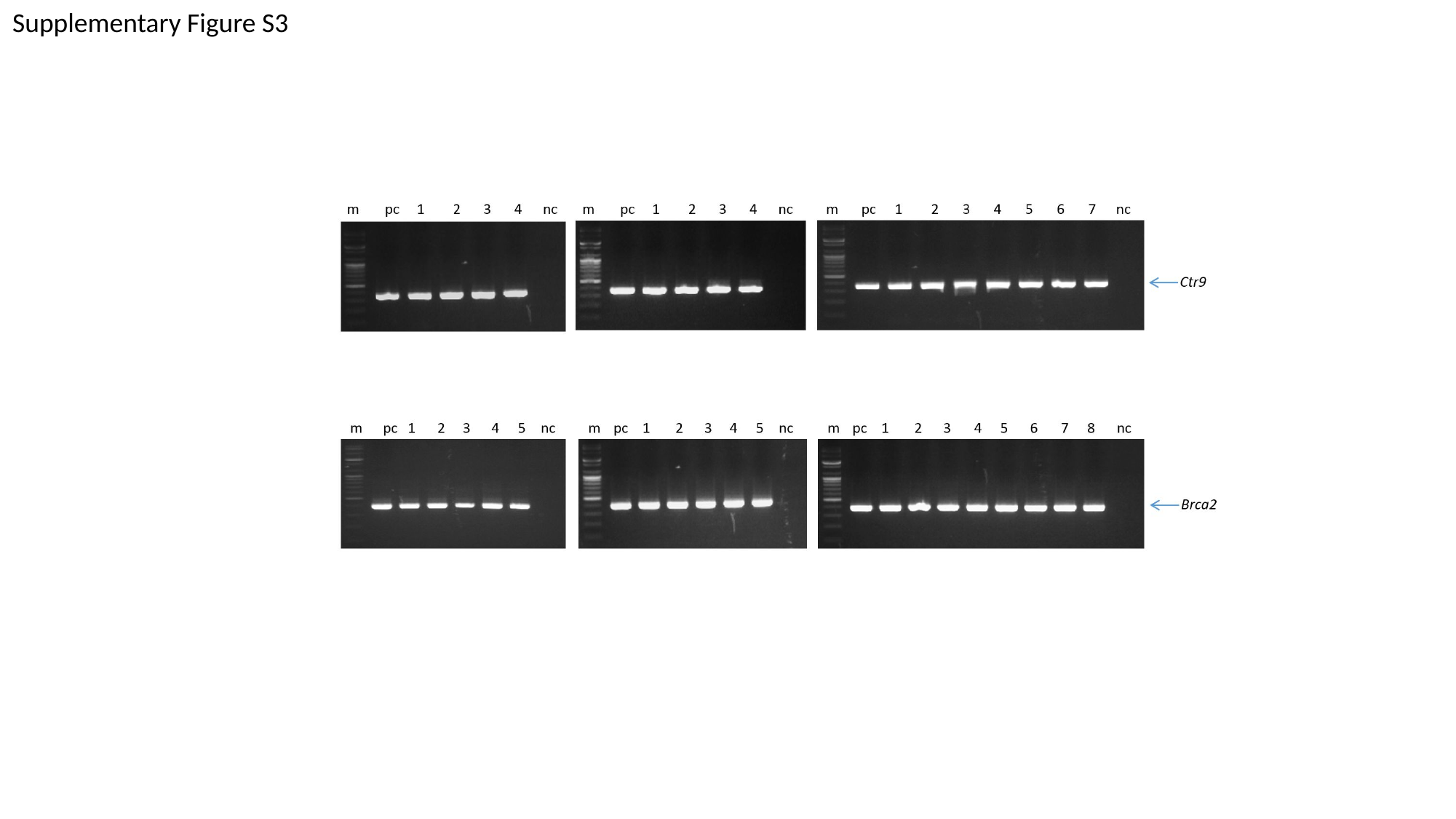

Supplementary Figure S3

## Slide 4
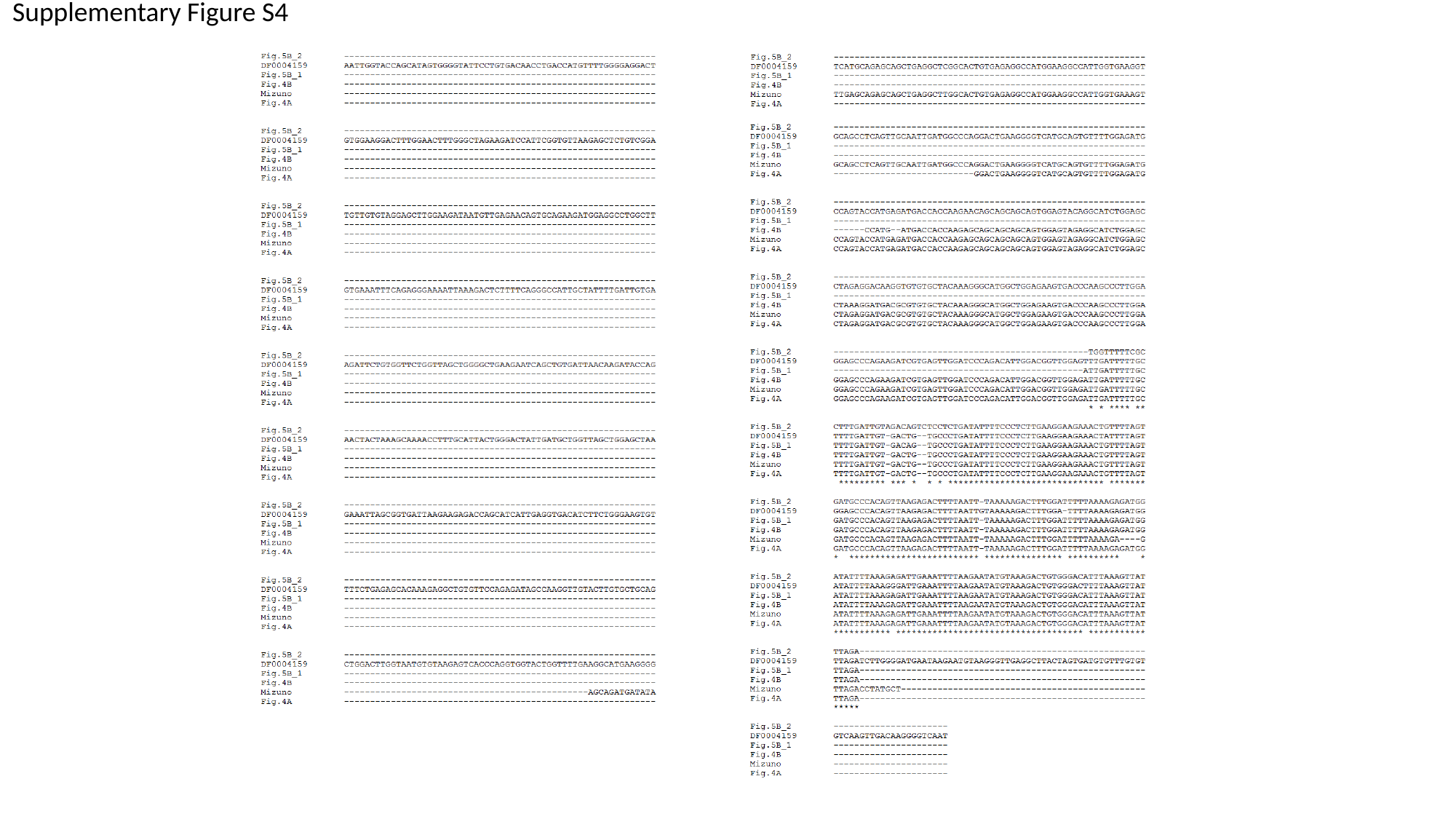

Supplementary Figure S4

## Slide 5
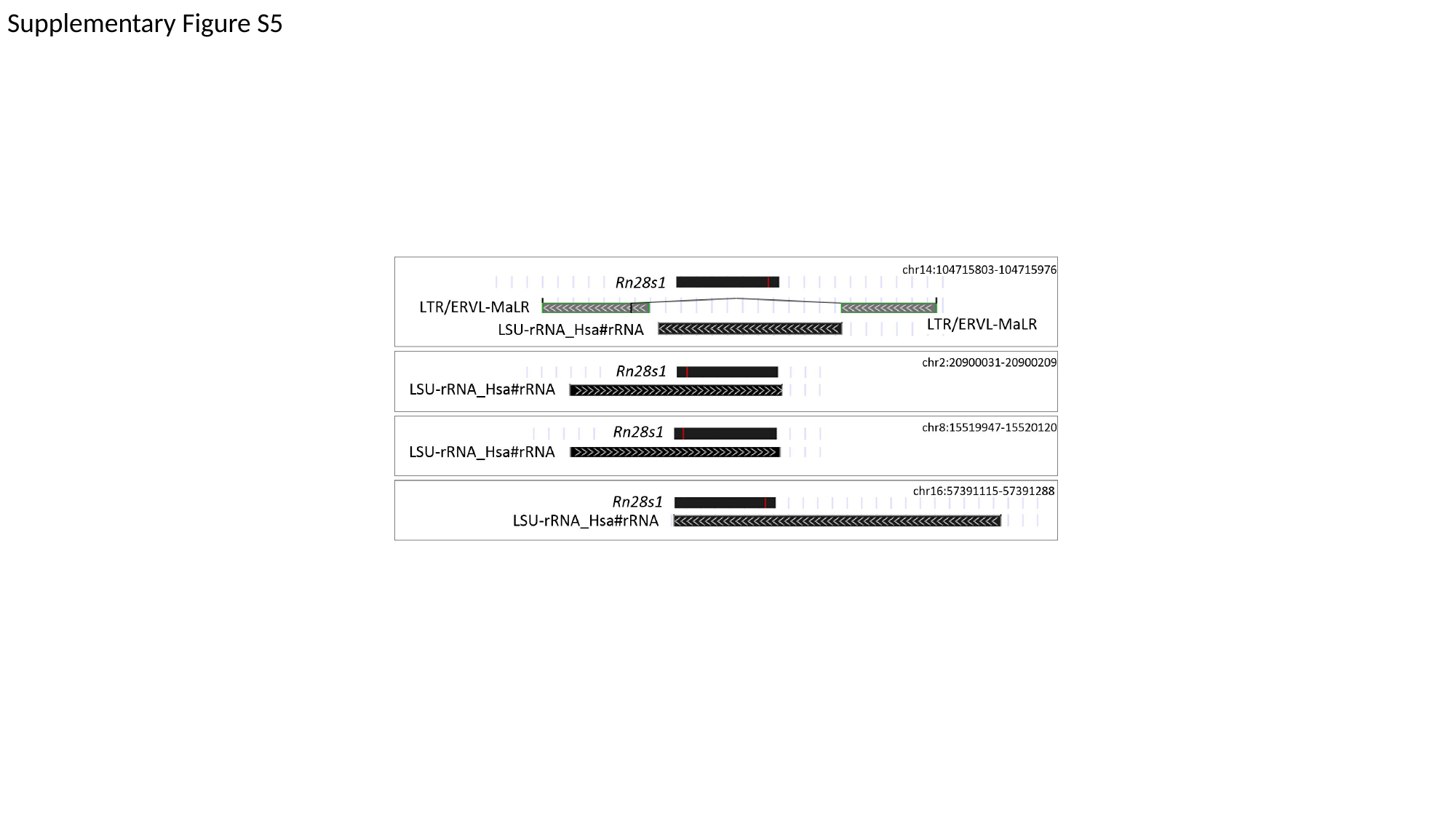

Supplementary Figure S5

## Slide 6
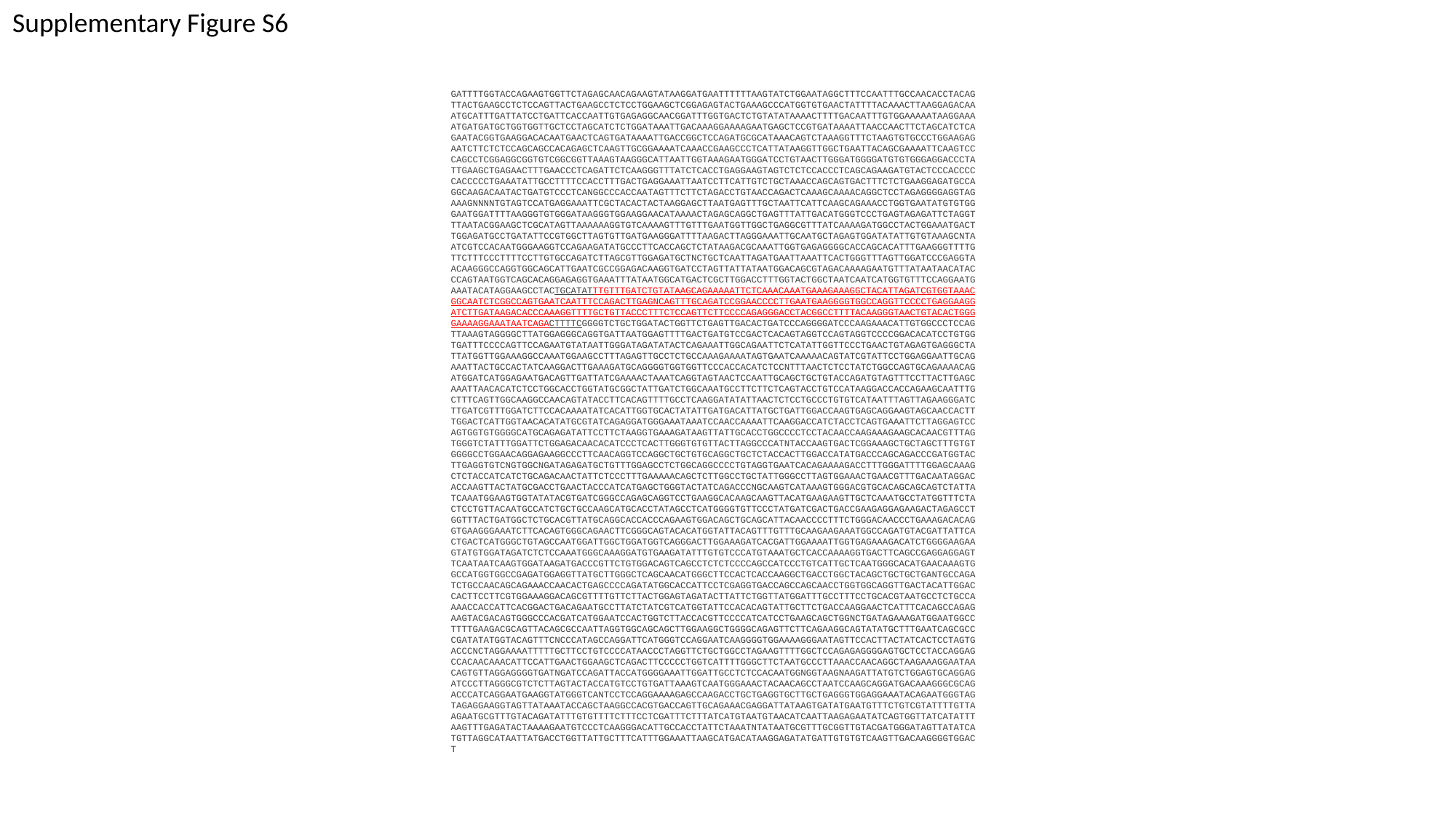

Supplementary Figure S6
GATTTTGGTACCAGAAGTGGTTCTAGAGCAACAGAAGTATAAGGATGAATTTTTTAAGTATCTGGAATAGGCTTTCCAATTTGCCAACACCTACAGTTACTGAAGCCTCTCCAGTTACTGAAGCCTCTCCTGGAAGCTCGGAGAGTACTGAAAGCCCATGGTGTGAACTATTTTACAAACTTAAGGAGACAAATGCATTTGATTATCCTGATTCACCAATTGTGAGAGGCAACGGATTTGGTGACTCTGTATATAAAACTTTTGACAATTTGTGGAAAAATAAGGAAAATGATGATGCTGGTGGTTGCTCCTAGCATCTCTGGATAAATTGACAAAGGAAAAGAATGAGCTCCGTGATAAAATTAACCAACTTCTAGCATCTCAGAATACGGTGAAGGACACAATGAACTCAGTGATAAAATTGACCGGCTCCAGATGCGCATAAACAGTCTAAAGGTTTCTAAGTGTGCCCTGGAAGAGAATCTTCTCTCCAGCAGCCACAGAGCTCAAGTTGCGGAAAATCAAACCGAAGCCCTCATTATAAGGTTGGCTGAATTACAGCGAAAATTCAAGTCCCAGCCTCGGAGGCGGTGTCGGCGGTTAAAGTAAGGGCATTAATTGGTAAAGAATGGGATCCTGTAACTTGGGATGGGGATGTGTGGGAGGACCCTATTGAAGCTGAGAACTTTGAACCCTCAGATTCTCAAGGGTTTATCTCACCTGAGGAAGTAGTCTCTCCACCCTCAGCAGAAGATGTACTCCCACCCCCACCCCCTGAAATATTGCCTTTTCCACCTTTGACTGAGGAAATTAATCCTTCATTGTCTGCTAAACCAGCAGTGACTTTCTCTGAAGGAGATGCCAGGCAAGACAATACTGATGTCCCTCANGGCCCACCAATAGTTTCTTCTAGACCTGTAACCAGACTCAAAGCAAAACAGGCTCCTAGAGGGGAGGTAGAAAGNNNNTGTAGTCCATGAGGAAATTCGCTACACTACTAAGGAGCTTAATGAGTTTGCTAATTCATTCAAGCAGAAACCTGGTGAATATGTGTGGGAATGGATTTTAAGGGTGTGGGATAAGGGTGGAAGGAACATAAAACTAGAGCAGGCTGAGTTTATTGACATGGGTCCCTGAGTAGAGATTCTAGGTTTAATACGGAAGCTCGCATAGTTAAAAAAGGTGTCAAAAGTTTGTTTGAATGGTTGGCTGAGGCGTTTATCAAAAGATGGCCTACTGGAAATGACTTGGAGATGCCTGATATTCCGTGGCTTAGTGTTGATGAAGGGATTTTAAGACTTAGGGAAATTGCAATGCTAGAGTGGATATATTGTGTAAAGCNTAATCGTCCACAATGGGAAGGTCCAGAAGATATGCCCTTCACCAGCTCTATAAGACGCAAATTGGTGAGAGGGGCACCAGCACATTTGAAGGGTTTTGTTCTTTCCCTTTTCCTTGTGCCAGATCTTAGCGTTGGAGATGCTNCTGCTCAATTAGATGAATTAAATTCACTGGGTTTAGTTGGATCCCGAGGTAACAAGGGCCAGGTGGCAGCATTGAATCGCCGGAGACAAGGTGATCCTAGTTATTATAATGGACAGCGTAGACAAAAGAATGTTTATAATAACATACCCAGTAATGGTCAGCACAGGAGAGGTGAAATTTATAATGGCATGACTCGCTTGGACCTTTGGTACTGGCTAATCAATCATGGTGTTTCCAGGAATGAAATACATAGGAAGCCTACTGCATATTTGTTTGATCTGTATAAGCAGAAAAATTCTCAAACAAATGAAAGAAAGGCTACATTAGATCGTGGTAAACGGCAATCTCGGCCAGTGAATCAATTTCCAGACTTGAGNCAGTTTGCAGATCCGGAACCCCTTGAATGAAGGGGTGGCCAGGTTCCCCTGAGGAAGGATCTTGATAAGACACCCAAAGGTTTTGCTGTTACCCTTTCTCCAGTTCTTCCCCAGAGGGACCTACGGCCTTTTACAAGGGTAACTGTACACTGGGGAAAAGGAAATAATCAGACTTTTCGGGGTCTGCTGGATACTGGTTCTGAGTTGACACTGATCCCAGGGGATCCCAAGAAACATTGTGGCCCTCCAGTTAAAGTAGGGGCTTATGGAGGGCAGGTGATTAATGGAGTTTTGACTGATGTCCGACTCACAGTAGGTCCAGTAGGTCCCCGGACACATCCTGTGGTGATTTCCCCAGTTCCAGAATGTATAATTGGGATAGATATACTCAGAAATTGGCAGAATTCTCATATTGGTTCCCTGAACTGTAGAGTGAGGGCTATTATGGTTGGAAAGGCCAAATGGAAGCCTTTAGAGTTGCCTCTGCCAAAGAAAATAGTGAATCAAAAACAGTATCGTATTCCTGGAGGAATTGCAGAAATTACTGCCACTATCAAGGACTTGAAAGATGCAGGGGTGGTGGTTCCCACCACATCTCCNTTTAACTCTCCTATCTGGCCAGTGCAGAAAACAGATGGATCATGGAGAATGACAGTTGATTATCGAAAACTAAATCAGGTAGTAACTCCAATTGCAGCTGCTGTACCAGATGTAGTTTCCTTACTTGAGCAAATTAACACATCTCCTGGCACCTGGTATGCGGCTATTGATCTGGCAAATGCCTTCTTCTCAGTACCTGTCCATAAGGACCACCAGAAGCAATTTGCTTTCAGTTGGCAAGGCCAACAGTATACCTTCACAGTTTTGCCTCAAGGATATATTAACTCTCCTGCCCTGTGTCATAATTTAGTTAGAAGGGATCTTGATCGTTTGGATCTTCCACAAAATATCACATTGGTGCACTATATTGATGACATTATGCTGATTGGACCAAGTGAGCAGGAAGTAGCAACCACTTTGGACTCATTGGTAACACATATGCGTATCAGAGGATGGGAAATAAATCCAACCAAAATTCAAGGACCATCTACCTCAGTGAAATTCTTAGGAGTCCAGTGGTGTGGGGCATGCAGAGATATTCCTTCTAAGGTGAAAGATAAGTTATTGCACCTGGCCCCTCCTACAACCAAGAAAGAAGCACAACGTTTAGTGGGTCTATTTGGATTCTGGAGACAACACATCCCTCACTTGGGTGTGTTACTTAGGCCCATNTACCAAGTGACTCGGAAAGCTGCTAGCTTTGTGTGGGGCCTGGAACAGGAGAAGGCCCTTCAACAGGTCCAGGCTGCTGTGCAGGCTGCTCTACCACTTGGACCATATGACCCAGCAGACCCGATGGTACTTGAGGTGTCNGTGGCNGATAGAGATGCTGTTTGGAGCCTCTGGCAGGCCCCTGTAGGTGAATCACAGAAAAGACCTTTGGGATTTTGGAGCAAAGCTCTACCATCATCTGCAGACAACTATTCTCCCTTTGAAAAACAGCTCTTGGCCTGCTATTGGGCCTTAGTGGAAACTGAACGTTTGACAATAGGACACCAAGTTACTATGCGACCTGAACTACCCATCATGAGCTGGGTACTATCAGACCCNGCAAGTCATAAAGTGGGACGTGCACAGCAGCAGTCTATTATCAAATGGAAGTGGTATATACGTGATCGGGCCAGAGCAGGTCCTGAAGGCACAAGCAAGTTACATGAAGAAGTTGCTCAAATGCCTATGGTTTCTACTCCTGTTACAATGCCATCTGCTGCCAAGCATGCACCTATAGCCTCATGGGGTGTTCCCTATGATCGACTGACCGAAGAGGAGAAGACTAGAGCCTGGTTTACTGATGGCTCTGCACGTTATGCAGGCACCACCCAGAAGTGGACAGCTGCAGCATTACAACCCCTTTCTGGGACAACCCTGAAAGACACAGGTGAAGGGAAATCTTCACAGTGGGCAGAACTTCGGGCAGTACACATGGTATTACAGTTTGTTTGCAAGAAGAAATGGCCAGATGTACGATTATTCACTGACTCATGGGCTGTAGCCAATGGATTGGCTGGATGGTCAGGGACTTGGAAAGATCACGATTGGAAAATTGGTGAGAAAGACATCTGGGGAAGAAGTATGTGGATAGATCTCTCCAAATGGGCAAAGGATGTGAAGATATTTGTGTCCCATGTAAATGCTCACCAAAAGGTGACTTCAGCCGAGGAGGAGTTCAATAATCAAGTGGATAAGATGACCCGTTCTGTGGACAGTCAGCCTCTCTCCCCAGCCATCCCTGTCATTGCTCAATGGGCACATGAACAAAGTGGCCATGGTGGCCGAGATGGAGGTTATGCTTGGGCTCAGCAACATGGGCTTCCACTCACCAAGGCTGACCTGGCTACAGCTGCTGCTGANTGCCAGATCTGCCAACAGCAGAAACCAACACTGAGCCCCAGATATGGCACCATTCCTCGAGGTGACCAGCCAGCAACCTGGTGGCAGGTTGACTACATTGGACCACTTCCTTCGTGGAAAGGACAGCGTTTTGTTCTTACTGGAGTAGATACTTATTCTGGTTATGGATTTGCCTTTCCTGCACGTAATGCCTCTGCCAAAACCACCATTCACGGACTGACAGAATGCCTTATCTATCGTCATGGTATTCCACACAGTATTGCTTCTGACCAAGGAACTCATTTCACAGCCAGAGAAGTACGACAGTGGGCCCACGATCATGGAATCCACTGGTCTTACCACGTTCCCCATCATCCTGAAGCAGCTGGNCTGATAGAAAGATGGAATGGCCTTTTGAAGACGCAGTTACAGCGCCAATTAGGTGGCAGCAGCTTGGAAGGCTGGGGCAGAGTTCTTCAGAAGGCAGTATATGCTTTGAATCAGCGCCCGATATATGGTACAGTTTCNCCCATAGCCAGGATTCATGGGTCCAGGAATCAAGGGGTGGAAAAGGGAATAGTTCCACTTACTATCACTCCTAGTGACCCNCTAGGAAAATTTTTGCTTCCTGTCCCCATAACCCTAGGTTCTGCTGGCCTAGAAGTTTTGGCTCCAGAGAGGGGAGTGCTCCTACCAGGAGCCACAACAAACATTCCATTGAACTGGAAGCTCAGACTTCCCCCTGGTCATTTTGGGCTTCTAATGCCCTTAAACCAACAGGCTAAGAAAGGAATAACAGTGTTAGGAGGGGTGATNGATCCAGATTACCATGGGGAAATTGGATTGCCTCTCCACAATGGNGGTAAGNAAGATTATGTCTGGAGTGCAGGAGATCCCTTAGGGCGTCTCTTAGTACTACCATGTCCTGTGATTAAAGTCAATGGGAAACTACAACAGCCTAATCCAAGCAGGATGACAAAGGGCGCAGACCCATCAGGAATGAAGGTATGGGTCANTCCTCCAGGAAAAGAGCCAAGACCTGCTGAGGTGCTTGCTGAGGGTGGAGGAAATACAGAATGGGTAGTAGAGGAAGGTAGTTATAAATACCAGCTAAGGCCACGTGACCAGTTGCAGAAACGAGGATTATAAGTGATATGAATGTTTCTGTCGTATTTTGTTAAGAATGCGTTTGTACAGATATTTGTGTTTTCTTTCCTCGATTTCTTTATCATGTAATGTAACATCAATTAAGAGAATATCAGTGGTTATCATATTTAAGTTTGAGATACTAAAAGAATGTCCCTCAAGGGACATTGCCACCTATTCTAAATNTATAATGCGTTTGCGGTTGTACGATGGGATAGTTATATCATGTTAGGCATAATTATGACCTGGTTATTGCTTTCATTTGGAAATTAAGCATGACATAAGGAGATATGATTGTGTGTCAAGTTGACAAGGGGTGGACT

## Slide 7
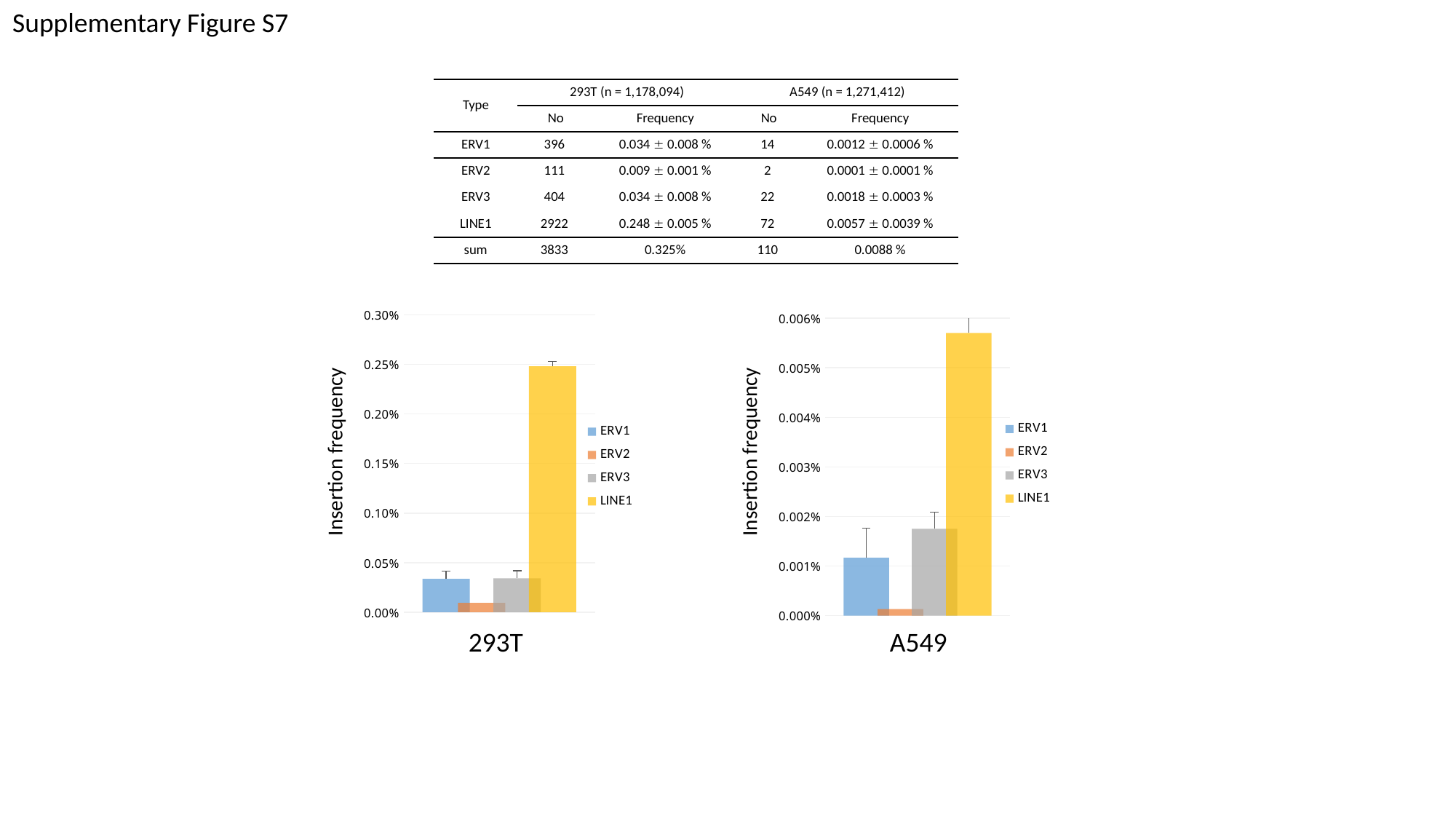

Supplementary Figure S7
| Type | 293T (n = 1,178,094) | | A549 (n = 1,271,412) | |
| --- | --- | --- | --- | --- |
| | No | Frequency | No | Frequency |
| ERV1 | 396 | 0.034 ± 0.008 % | 14 | 0.0012 ± 0.0006 % |
| ERV2 | 111 | 0.009 ± 0.001 % | 2 | 0.0001 ± 0.0001 % |
| ERV3 | 404 | 0.034 ± 0.008 % | 22 | 0.0018 ± 0.0003 % |
| LINE1 | 2922 | 0.248 ± 0.005 % | 72 | 0.0057 ± 0.0039 % |
| sum | 3833 | 0.325% | 110 | 0.0088 % |
### Chart
| Category | ERV1 | ERV2 | ERV3 | LINE1 |
|---|---|---|---|---|
| 293T | 0.0003361322728995134 | 9.37778189393308e-05 | 0.0003418560200604105 | 0.002481050027122091 |Insertion frequency
293T
### Chart
| Category | ERV1 | ERV2 | ERV3 | LINE1 |
|---|---|---|---|---|
| A549 | 1.1688975434869059e-05 | 1.3342783240502528e-06 | 1.750015587535154e-05 | 5.7034992430284236e-05 |A549
Insertion frequency

## Slide 8
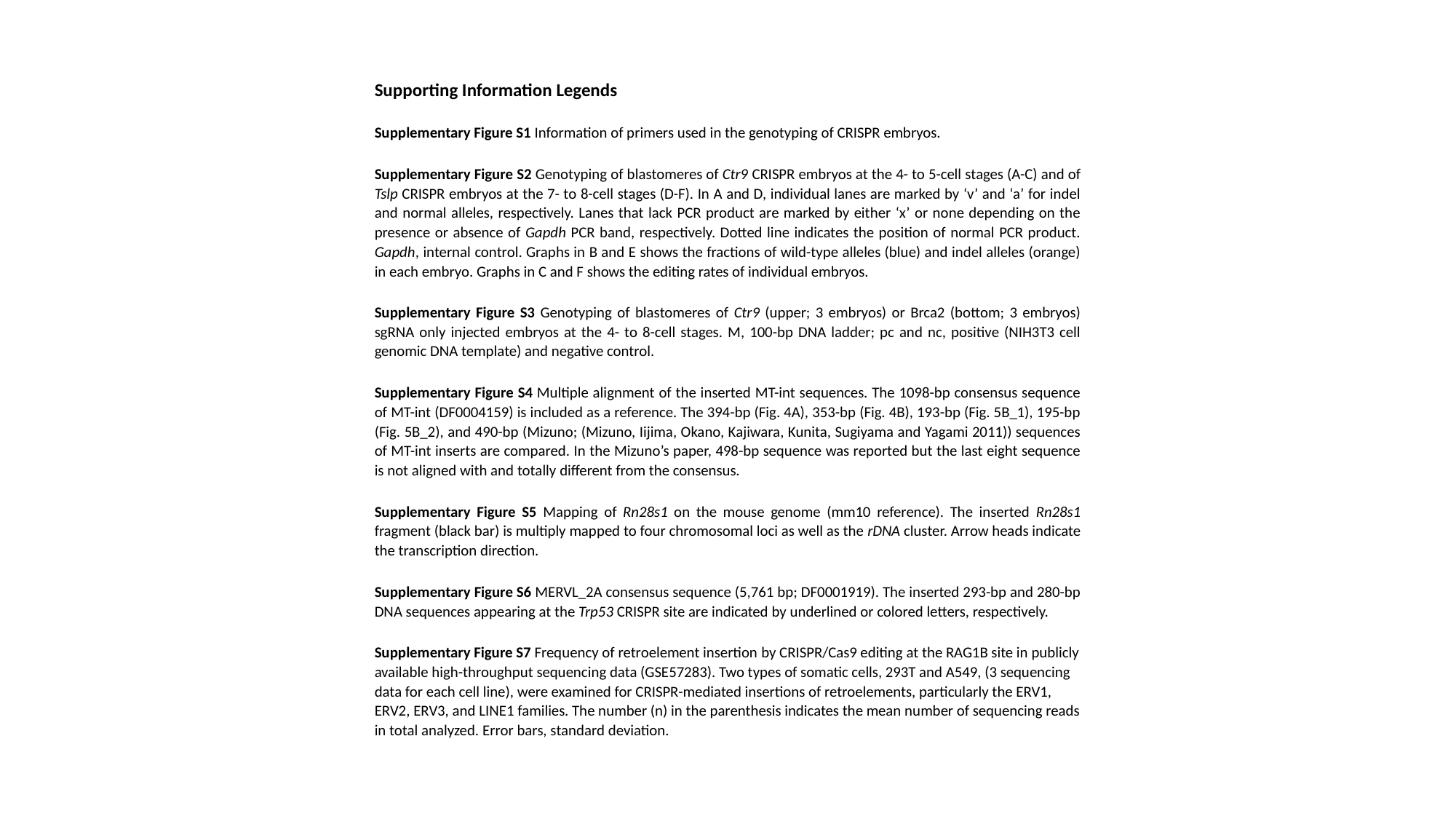

Supporting Information Legends
Supplementary Figure S1 Information of primers used in the genotyping of CRISPR embryos.
Supplementary Figure S2 Genotyping of blastomeres of Ctr9 CRISPR embryos at the 4- to 5-cell stages (A-C) and of Tslp CRISPR embryos at the 7- to 8-cell stages (D-F). In A and D, individual lanes are marked by ‘v’ and ‘a’ for indel and normal alleles, respectively. Lanes that lack PCR product are marked by either ‘x’ or none depending on the presence or absence of Gapdh PCR band, respectively. Dotted line indicates the position of normal PCR product. Gapdh, internal control. Graphs in B and E shows the fractions of wild-type alleles (blue) and indel alleles (orange) in each embryo. Graphs in C and F shows the editing rates of individual embryos.
Supplementary Figure S3 Genotyping of blastomeres of Ctr9 (upper; 3 embryos) or Brca2 (bottom; 3 embryos) sgRNA only injected embryos at the 4- to 8-cell stages. M, 100-bp DNA ladder; pc and nc, positive (NIH3T3 cell genomic DNA template) and negative control.
Supplementary Figure S4 Multiple alignment of the inserted MT-int sequences. The 1098-bp consensus sequence of MT-int (DF0004159) is included as a reference. The 394-bp (Fig. 4A), 353-bp (Fig. 4B), 193-bp (Fig. 5B_1), 195-bp (Fig. 5B_2), and 490-bp (Mizuno; (Mizuno, Iijima, Okano, Kajiwara, Kunita, Sugiyama and Yagami 2011)) sequences of MT-int inserts are compared. In the Mizuno’s paper, 498-bp sequence was reported but the last eight sequence is not aligned with and totally different from the consensus.
Supplementary Figure S5 Mapping of Rn28s1 on the mouse genome (mm10 reference). The inserted Rn28s1 fragment (black bar) is multiply mapped to four chromosomal loci as well as the rDNA cluster. Arrow heads indicate the transcription direction.
Supplementary Figure S6 MERVL_2A consensus sequence (5,761 bp; DF0001919). The inserted 293-bp and 280-bp DNA sequences appearing at the Trp53 CRISPR site are indicated by underlined or colored letters, respectively.
Supplementary Figure S7 Frequency of retroelement insertion by CRISPR/Cas9 editing at the RAG1B site in publicly available high-throughput sequencing data (GSE57283). Two types of somatic cells, 293T and A549, (3 sequencing data for each cell line), were examined for CRISPR-mediated insertions of retroelements, particularly the ERV1, ERV2, ERV3, and LINE1 families. The number (n) in the parenthesis indicates the mean number of sequencing reads in total analyzed. Error bars, standard deviation.
